# Urinary tract infections in patients of University Hospital Center of Tirana

**DOI:** 10.1186/1753-6561-5-S6-P202

**Published:** 2011-06-29

**Authors:** G Kasmi, S Bino, I Kasmi, S Tafai, A Simaku

**Affiliations:** 1 Laboratory of Microbiology, Faculty of Medicine, Tirana, Albania; 2 Infectious diseases, Institute of Public Health, Tirana, Albania; 3 Pediatric , University Hospital Center, Tirana, Albania; 4 Laboratory of Microbiology, Hospital "Shefqet Ndroqi", Tirana, Albania

## Introduction / objectives

Urinary tract infections (UTIs) are the most common type of nosocomial infections. The majority of nosocomial UTIs occur following instrumentation. Because nearly 10% of all hospitalized patients are catheterized, preventing nosocomial UTIs is a major factor in decreasing nosocomial infections. The aim of the study was to register the prevalence, etiology and antimicrobial susceptibility of nosocomial urinary tract infection pathogens isolated in UHC.

## Methods

It was a cross-sectional study. In one day, a total of 893 urine samples were taken from hospitalized patients of UHC. The Vitek 2 automated system was used to identify and to detect antibiotic susceptibility. We collected data regarding etiology and antimicrobial resistance profile of the urinary isolates collected.

## Results

The six most commonly isolated organisms were in decreasing order: E*.coli*, *Candida sp*, *P.aeruginosa*, *E.cloacae*, *Klebsiella* sp and *Enterococcus sp*.

The overall resistance rate to ampicillin in Gram - negatives was 88%.

The antimicrobial resistance patterns of the study isolates confirm the changes reported in nosocomial pathogens from other sources.

## Conclusion

The prevalence rate of nosocomial UTIs was 18.9 %. These data show the high level of antimicrobial resistance amongst the uropathogens causing nosocomial UTIs. UTIs is related to the use of indwelling urinary catheters and other intravesical procedures. The levels of resistance of pathogens must be a clear reason for stricter guidelines and regulations in antimicrobial policy.

## Disclosure of interest

None declared.

